# Comparison of Machine Learning Models for Prediction of Initial Intravenous Immunoglobulin Resistance in Children With Kawasaki Disease

**DOI:** 10.3389/fped.2020.570834

**Published:** 2020-12-03

**Authors:** Yasutaka Kuniyoshi, Haruka Tokutake, Natsuki Takahashi, Azusa Kamura, Sumie Yasuda, Makoto Tashiro

**Affiliations:** Department of Pediatrics, Tsugaruhoken Medical COOP Kensei Hospital, Hirosaki, Japan

**Keywords:** area under the curve, extreme gradient boosting, support vector machine, logistic regression, nested cross-validation, predictive model

## Abstract

We constructed an optimal machine learning (ML) method for predicting intravenous immunoglobulin (IVIG) resistance in children with Kawasaki disease (KD) using commonly available clinical and laboratory variables. We retrospectively collected 98 clinical records of hospitalized children with KD (2–109 months of age). We found that 20 (20%) children were resistant to initial IVIG therapy. We trained three ML techniques, including logistic regression, linear support vector machine, and eXtreme gradient boosting with 10 variables against IVIG resistance. Moreover, we estimated the predictive performance based on nested 5-fold cross-validation (CV). We also selected variables using the recursive feature elimination method and performed the nested 5-fold CV with selected variables in a similar manner. We compared ML models with the existing system regardless of their predictive performance. Results of the area under the receiver operator characteristic curve were in the range of 0.58–0.60 in the all-variable model and 0.60–0.75 in the select model. The specificities were more than 0.90 and higher than those in existing scoring systems, but the sensitivities were lower. Three ML models based on demographics and routine laboratory variables did not provide reliable performance. This is possibly the first study that has attempted to establish a better predictive model. Additional biomarkers are probably needed to generate an effective prediction model.

## Introduction

In developed countries, Kawasaki disease (KD) is the major cause of acquired heart disease in children (1). The main complication of KD is coronary artery abnormality (CAA) due to systemic vasculitis (1). The effectiveness of high-dose intravenous immunoglobulin (IVIG) therapy has been established as an initial KD treatment (2). However, approximately 10–20% children with KD are refractory to this treatment and develop persistent or recurrent fever after initial IVIG therapy (3, 4). IVIG resistance is a risk factor for the occurrence of CAA (5). Moreover, the development of a more effective treatment options has been challenging. The American Heart Association has reported that patients who were predicted to be at a high risk for development of CAA may benefit from primary adjunctive therapy such as IVIG and corticosteroids (2). Therefore, developing a reliable tool for predicting IVIG resistance is important to reduce the occurrence of CAA.

Several scoring systems (6–12) have been proposed. However, the predictive capacity of the existing scoring systems may not be sufficient, and some scoring systems have poor predictive performance for external datasets (13–15). Machine learning (ML) techniques have been applied to clinical diagnosis and prognosis prediction in many fields of medicine (16). To the best of our knowledge, few studies have applied ML methods for predicting resistance to initial IVIG therapy in patients with KD (17). We aimed to construct an optimal ML method for predicting IVIG resistance in children with KD using commonly available clinical and laboratory variables.

## Materials and Methods

### Patients and Data Collection

We retrospectively collected clinical records of patients with KD who were diagnosed based on the Japanese diagnostic guidelines for KD (18) and hospitalized at Tsugaruhoken Medical COOP Kensei Hospital between January 2010 and October 2019. Patients diagnosed with KD presented with minimum five of the six major symptoms, including fever. Patients with only four or less major symptoms and those with CAA were not included. We excluded children who received initial IVIG treatment ≥10 days after the onset and children administered initial doses of <2 g/kg/day. We defined the first illness day as the first day on which a patient had fever. We defined a responder as a patient whose temperature had decreased to <37.5°C within 36 h after initial IVIG treatment (9, 15).

We collected the following data before the initial IVIG treatment: months of age, gender, illness days with IVIG administration, white blood cell count (WBC), neutrophil percentage, hematocrit (Ht), platelet count (PLT), aspartate aminotransferase (AST), alanine aminotransferase (ALT), total bilirubin (TBil), and sodium (Na), albumin (Alb), and C-reactive protein (CRP) levels. All these variables were available before treatment.

We defined coronary arteries as abnormal when the luminal diameters were more than 3.0 mm in children younger than 5 years or more than 4.0 mm in those 5 years and older, when the internal diameter of a segment was 1.5 times or greater than that of an adjacent segment, or when the luminal contour was evidently irregular (19). We recorded the maximum coronary artery diameter within 1 month after the onset of the disease.

### Statistical Analysis

We performed statistical analyses using Python version 3.6 (Python Software Foundation). We applied Mann–Whitney *U*-tests for continuous variables and Chi-square tests for categorical variables.

We evaluated the predictive performance of the three supervised ML classifiers and existing scoring systems. We trained logistic regression (LR) with L2 regularization, linear support vector machine (SVM), and eXtreme gradient boosting (XGB) models to predict IVIG resistance, using scikit-learn and XGBoost packages. We evaluated the predictive performance based on sensitivity, specificity, and area under the receiver operator characteristic curve (AUC). We produced three ML models with 10 variables that did not contain missing values (months of age, gender, illness days with IVIG administration, WBC, Ht, PLT, AST, Na, Alb, and CRP).

**Figure 1:**
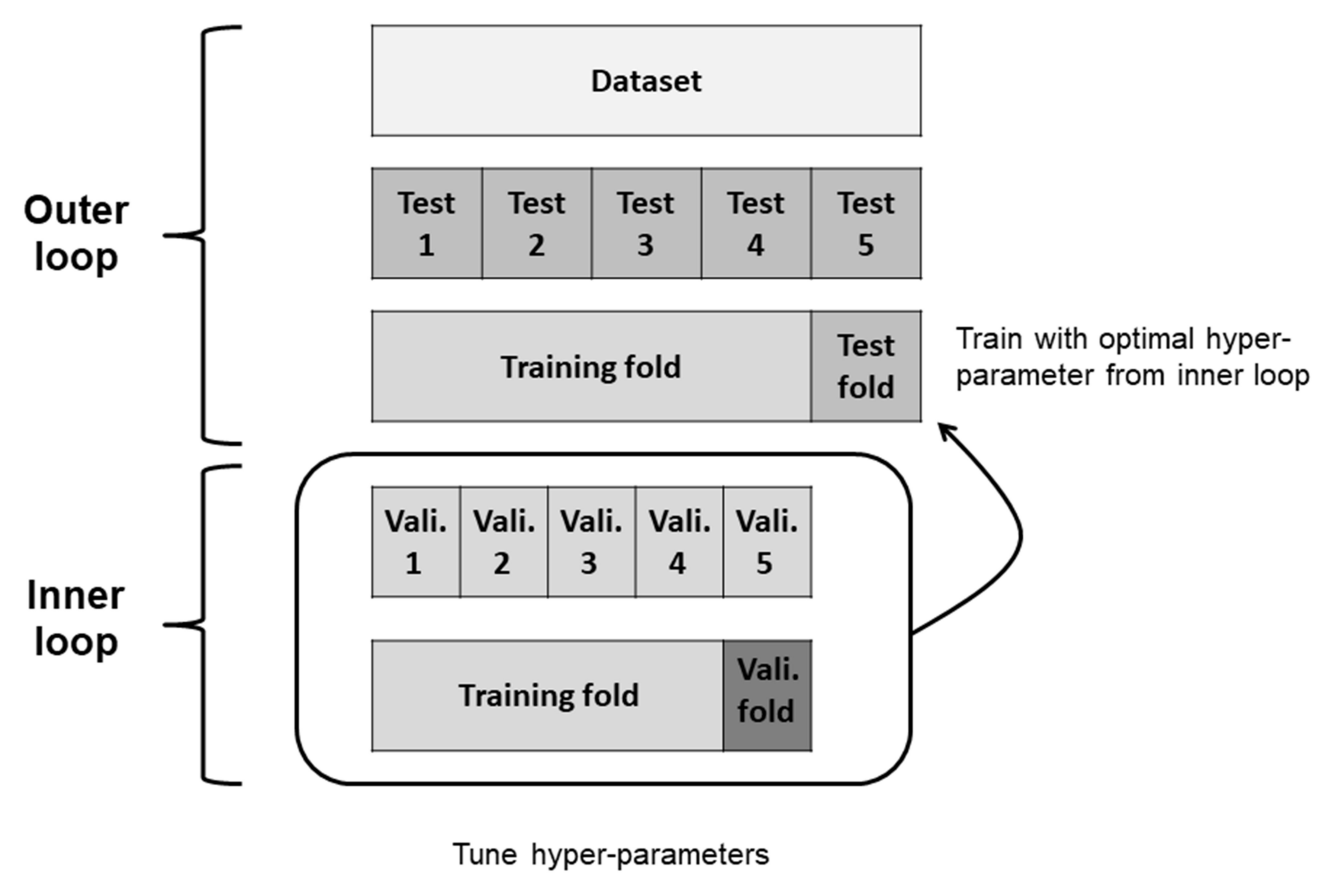
Flow chart of the 5-fold nested cross-validation. Vali., validation.

To evaluate the predictive performance of the three ML models and algorithms, we used the nested 5-fold cross-validation (CV) approach (20) with *GridSearchCV* for hyper-parameter optimization. We applied a nested CV procedure to estimate an unbiased generalization performance of ML algorithms (21). The two CV cycles included an inner loop for tuning hyper-parameters and outer loop for estimating performance in nested CV (Figure 1). First, the original dataset was divided into five data folds with approximately equal numbers of respondent and non-respondent cases. One data fold was reserved for *test fold*. The remaining four data folds (*training folds*) were passed to the inner loop. The inner loop performed 5-fold CVs to identify the best hyper-parameter combination. We selected the hyper-parameter combination that maximized each performance metrics over all steps of the inner loop. In LR and linear SVM models, the penalty parameter *C* was explored in [0.01, 0.1, 1, 10, and 100]. In XGB model, the maximum depth of a tree (max_depth), the minimum sum of instance weight needed in a child (min_child_weight), and gamma were explored in [3, 5], [1, 2, 3], and [0, 3, 10], respectively. For the other hyper-parameters, we used default values of the scikit-learn method. These were tuned by testing all possible hyper-parameter combinations in the inner CV. We then trained our model on *training folds* using the best hyper-parameter combination; thereafter, we evaluated model performance on the *test fold*. This process was repeated five times, once for each iteration of the outer loop. Finally, we calculated the average performance over 5-folds. We also repeated nested CVs 10 times in separate splits and derived the average of the results to avoid sampling bias and data overfitting.

Additionally, we selected variables using the recursive feature elimination method. Then, we performed a nested 5-fold CV with selected variables in a similar manner. In all, we have constructed and then evaluated two types of models: all-variable model and select-variable model.

## Results

### Characteristics of Patients

We collected data from 109 children with KD treated at our hospital. We excluded data from 11 children because 9 children had received initial IVIG at <2 g/kg/day and 4 had received initial IVIG treatment ≥10 days after the onset of the disease. Consequently, we statistically analyzed data from 98 children aged 2–109 months. Table 1 summarizes the demographic and laboratory data of patients. Among them, 20 (20%) children were resistant to the initial IVIG therapy. Only the AST and ALT levels were significantly higher in the IVIG-responsive group than in the IVIG resistant group. The proportion of CAA in the IVIG resistant group was higher than that in the IVIG-responsive group.

**Table 1:** Comparison of clinical and laboratory characteristics in IVIG-responsive and -resistant patients.

	**Responsive** **(*n* = 78)**	**Resistant** **(*n* = 20)**	***P*-value**
Age, months of age, median (IQR)	22 (9–37)	26 (17–30)	0.49
Illness days with IVIG administration, days, median (IQR)	5 (4–6)	4 (3.8–5)	0.16
Gender, male, *n* (%)	40 (51)	13 (65)	0.40
White blood cell count, × 10^2^/mm^3^, median (IQR)	151 (121–175)	144 (113–179)	0.96
Neutrophil, %, median (IQR)	66 (59–76)	73 (67–79)	0.12
Hematocrit, %, median (IQR)	34 (32–36)	35 (33–36)	0.65
Platelet count, × 10^4^/mm^3^, median (IQR)	35 (28–42)	32 (27–38)	0.41
Aspartate aminotransferase, IU/L, median (IQR)	30 (24–43)	96 (34–308)	<0.001
Alanine aminotransferase, IU/L, median (IQR)	20 (12–32)	75 (20–232)	0.004
Total bilirubin, mg/dl, median (IQR)	0.53 (0.41–0.69)	0.81 (0.50–1.37)	0.36
Sodium, mmol/L, median (IQR)	133 (131–134)	132 (131–134)	0.88
Albumin, g/dl, median (IQR)	3.3 (3.1–3.6)	3.4 (3.1–3.6)	0.85
C-reactive protein, mg/dl, median (IQR)	6.3 (3.8–9.3)	7.4 (5.3–10.6)	0.26
Coronary artery abnormalities, *n* (%)	5 (6.4)	6 (30)	× 0.007

*IVIG, intravenous immunoglobulin; SD, standard deviation; IQR; interquartile range*.

*Data are analyzed by Mann–Whitney U tests for continuous variables and Chi-square tests for categorical variables*.

### Predictive Performance of the ML Model

As shown in Table 2, the AUCs of the all-variable models were 0.58–0.60 in all models, and those of the select-variable models were 0.60–0.75. The results on specificity and accuracy were 0.94–0.99 and 0.78–0.79 in the all-variable models, and 0.96–1.00 and 0.78–0.80 in the select-variable models. The results of specificity and accuracy were high, but those on sensitivity were all lower.

**Table 2:** Prediction performances of the three machine learning models and existing scoring systems.

			**Feature**	**AUC**	**Sensitivity**	**Specificity**	**Accuracy**
All-variable model	LR		All 10 variables	0.59 ± 0.052	0.22 ± 0.055	0.94 ± 0.017	0.79 ± 0.021
	Linear SVM		All 10 variables	0.58 ± 0.040	0.20 ± 0.059	0.95 ± 0.014	0.79 ± 0.018
	XGBoost		All 10 variables	0.60 ± 0.048	0.26 ± 0.095	0.99 ± 0.021	0.78 ± 0.026
Select-variable model	LR	Model 1	AST	0.75 ± 0.011	0.15 ± 0.039	0.97 ± 0.006	0.79 ± 0.012
		Model 2	WBC, AST	0.67 ± 0.027	0.16 ± 0.037	0.97 ± 0.008	0.80 ± 0.011
		Model 3	Day, WBC, PLT, AST, CRP	0.67 ± 0.022	0.19 ± 0.049	0.96 ± 0.049	0.80 ± 0.010
	SVM	Model 1	AST	0.75 ± 0.011	0.14 ± 0.037	0.96 ± 0.012	0.79 ± 0.015
		Model 2	WBC, Ht, PLT, AST	0.66 ± 0.035	0.16 ± 0.039	0.97 ± 0.010	0.80 ± 0.010
		Model 3	WBC, AST	0.68 ± 0.024	0.14 ± 0.035	0.97 ± 0.006	0.79 ± 0.007
	XGBoost	Model 1	Na, AST	0.65 ± 0.032	0.28 ± 0.078	1.00 ± 0.008	0.78 ± 0.021
		Model 2	Age, Day, Ht, Na, AST, CRP	0.61 ± 0.036	0.31 ± 0.073	0.99 ± 0.008	0.79 ± 0.025
		Model 3	Age, Day, Ht, Na, AST, Alb, CRP	0.60 ± 0.035	0.33 ± 0.078	0.99 ± 0.008	0.79 ± 0.022
Existing scoring systems	Kobayashi (8) system (*n* = 93)			NA	0.70	0.62	0.63
	Egami (9) system (*n* = 98)			NA	0.55	0.81	0.76
	Sano (10) system (*n* = 62)			NA	0.41	0.96	0.81

*AUC, area under the receiver operator characteristic curve; LR, logistic regression; SVM, support vector machine; XGB, eXtreme gradient boosting; Age, months of age; Day, illness days with IVIG administration; WBC, white blood cell count; Ht, hematocrit; PLT, platelet count; AST, aspartate aminotransferase; Na, sodium; Alb, albumin; CRP, C-reactive protein; NA, not applicable*.

## Discussion

We retrospectively evaluated the performances of three ML models to predict the resistance to initial IVIG therapy in a single-center pediatric population of KD. Our results revealed that the three ML models based on demographics and routine laboratory variables did not perform reliably.

Different clinical scoring systems have been established to predict IVIG resistance, including those by Kobayashi et al. (6), Egami et al. (7), and Sano et al. (8) in Japan. The sensitivities and specificities of those systems were reported to be 0.76–0.78 and 0.76–0.86 in the original studies. However, almost all clinical scores published are limited in their predictive capacity. Similar predictive accuracies were not achieved in other populations (13–15). As shown in Table 2, the existing scoring systems also did not achieve a good prediction against our dataset.

Additional clinical information may be needed to improve the prediction model. Owing to the similarity of each clinical and laboratory characteristics between IVIG-responsive and -resistant patients in the current dataset, neither our model nor the existing model may have performed reliably. There may be a need to construct and evaluate new models that also incorporate clinical major symptoms (10) and/or other laboratory data such as erythrocyte sedimentation rate (10) or N-terminal pro-brain natriuretic peptide (22).

Our prediction models using three ML techniques have equally less reliable performance as the existing scoring systems; particularly, the sensitivity were low in all ML algorithms. Our results serve as a first step to establish a good prediction tool. Feature engineering or ensemble learning, which combines several ML techniques into one predictive model, may help improve performance. Alternatively, ML models have advantages over the existing prediction scoring systems. The predictive performances of scoring systems could differ depending on countries or ethnicities (11, 13, 23). ML is flexible and can be suitable for many tasks. Therefore, the ML approach makes it easy for the model to retrain and update the using the newest data.

To our best knowledge, this is the first study to compare the performances of ML methods for predicting IVIG resistance. There is a study which was designed to develop the prediction model using random forest (17). However, validation procedures were not conducted, though the performance was excellent. Conversely, there are certain limitations. First, the dataset was relatively small. However, we used nested CVs to obtain unbiased estimates of the true error. We also repeated the nested CVs 10 times and averaged the validation error to reduce sampling bias. Nested CV can choose the classification model by obtaining reliable classification performance and avoiding overfitting (24). Second, the present study was conducted based on dataset derived from a single center. Accordingly, our results may not apply to other populations. However, we consider it meaningful to rebuild the model, similarly using the center's original data. Third, this is a retrospective study. We need to perform a combined analysis of three ML models on a prospective basis.

In conclusion, we evaluated the performance of ML models for predicting resistance to IVIG therapy in children with KD. However, our three ML models based only on demographics and routine laboratory variables did not provide reliable performances. Further studies are needed to improve predictive models. Additional biomarkers are likely to be needed to generate an effective prediction model.

## Data Availability Statement

The raw data supporting the conclusions of this article will be made available by the authors, Yasutaka Kuniyoshi, upon reasonable request.

## Ethics Statement

The studies involving human participants were reviewed and approved by Ethics Committee of Tsugaruhoken Medial COOP Kensei Hospital. Written informed consent from the participants' legal guardian/next of kin was not required to participate in this study in accordance with the national legislation and the institutional requirements.

## Author Contributions

YK designed the study, drafted the manuscript, performed the statistical analysis, and interpreted the results. All authors have read and approved the final manuscript and contributed to data collection.

## Conflict of Interest

The authors declare that the research was conducted in the absence of any commercial or financial relationships that could be construed as a potential conflict of interest.

## Abbreviations

Alb: albumin
ALT: alanine aminotransferase
AST: aspartate aminotransferase
AUC: area under the receiver operator characteristic curve
CAA: coronary artery abnormality
CRP: C-reactive protein
CV: cross-validation
Ht: hematocrit
IVIG: intravenous immunoglobulin
KD: Kawasaki disease
LR: logistic regression
ML: machine learning
Na: sodium
PLT: platelet count
SVM: support vector machine
TBil: total bilirubin
WBC: white blood cell count
XGB: eXtreme gradient boosting.
